# Polyphenols from Mediterranean Plants: Biological Activities for Skin Photoprotection in Atopic Dermatitis, Psoriasis, and Chronic Urticaria

**DOI:** 10.3390/plants12203579

**Published:** 2023-10-15

**Authors:** Eleonora Di Salvo, Sebastiano Gangemi, Claudia Genovese, Nicola Cicero, Marco Casciaro

**Affiliations:** 1 Department of Biomedical and Dental Sciences and Morphofunctional Imaging, University of Messina, 98168 Messina, Italy; edisalvo@unime.it; 2 School and Operative Unit of Allergy and Clinical Immunology, Department of Clinical and Experimental Medicine, University of Messina, 98125 Messina, Italy; gangemis@unime.it (S.G.); marco.casciaro@unime.it (M.C.); 3 National Research Council, Institute for Agricultural and Forest Systems in the Mediterranean, Via Empedocle 58, 95128 Catania, Italy; claudia.genovese@cnr.it; 4 Science4Life, Spin Off Company, University of Messina, 98168 Messina, Italy

**Keywords:** polyphenols, food, skin, skin photoprotection, atopic dermatitis, psoriasis, chronic urticaria

## Abstract

Polyphenols are a diverse class of natural compounds that are widely distributed in various fruits, vegetables, and herbs. They possess antioxidant and anti-inflammatory properties and bring benefits in the prevention and treatment of various diseases. Studies suggested that polyphenols may improve cardiovascular health and may have neuroprotective effects. The Mediterranean region is a vast area. Although the territory encompasses a wide variety of cultures and dietary patterns, there are some commonalities in terms of the plant-based foods and their polyphenol content. Such polyphenols have been studied for their potential photoprotective effects on the skin. We focused on nutraceutical effects of Mediterranean plants in skin photoprotection in atopic dermatitis, psoriasis, and chronic urticaria. Results highlight the importance of exploring natural compounds for therapeutic purposes. The wide variety of polyphenols found in different foods and plants allows for a diverse range of pharmacological effects. The Mediterranean diet, rich in polyphenol-containing foods, is associated with a lower incidence of various chronic diseases, including dermatological conditions. While more research is needed to fully understand the mechanisms of action and optimal dosing of polyphenols, there is initial evidence to support their potential use as adjunctive therapy for atopic dermatitis, psoriasis, and chronic urticaria.

## 1. Introduction

Polyphenols are a diverse class of natural compounds that are widely distributed in various fruits, vegetables, and herbs. These compounds are characterized by the presence of multiple phenol units and possess potent antioxidant and anti-inflammatory properties. In recent years, several different types of polyphenols have been identified, each with its unique chemical structure and potential pharmacological functions [1,2,3]. One prominent class of polyphenols is the flavonoids. Flavonoids are a large and diverse group of compounds, which are widely distributed in plants. They can be further classified into different subtypes, including flavones, flavonols, flavanones, and anthocyanins [4,5]. Flavones, such as apigenin and luteolin, have been shown to possess anti-inflammatory and antioxidant properties, as well as a reduced risk of cancer and cardiovascular disease. Flavonols, such as quercetin and kaempferol, have also been linked to anti-inflammatory, antioxidant, and anti-cancer properties [6,7]. In addition, flavonols were linked to ameliorated cardiovascular health, including lower blood pressure and improved lipid levels. Flavanones, abundant in citrus fruits, have also been shown to exhibit anti-inflammatory and antioxidant activities. They have been linked to improved insulin sensitivity and a reduced risk of cardiovascular disease [8,9]. Similarly, anthocyanins, which give fruits and vegetables their red, purple, and blue colors, have potent antioxidant and anti-inflammatory properties. Anthocyanins were associated with improved cognitive function and a reduced risk of cardiovascular disease [10,11]. Another significant class of polyphenols is the stilbenoids, with resveratrol being the most extensively studied member. Resveratrol is found in grapes and has demonstrated anti-inflammatory and antioxidant properties, as well as a reduced risk of cancer and cardiovascular disease. Resveratrol has also been shown to improve insulin sensitivity and may have neuroprotective effects [12,13,14]. Phenolic acids, including caffeic acid and rosmarinic acid, are another class of polyphenols with potent antioxidant and anti-inflammatory properties. These compounds have been linked to a reduced risk of chronic diseases such as cancer, cardiovascular disease, and neurodegenerative disorders. Lignans are polyphenols found in flaxseed, sesame seeds, and whole grains, and they too possess anti-inflammatory and antioxidant properties. Lignans have been associated with a reduced risk of breast and prostate cancer and cardiovascular disease, and improved insulin sensitivity [15,16,17]. Other types of polyphenols include tannins, which are found in tea and wine, and ellagitannins, which are found in berries and nuts. Tannins possess potent antioxidant properties and may also have anti-inflammatory and anti-cancer effects [18]. Ellagitannins were reported as having anti-inflammatory and antioxidant activities, together with potential anti-cancer properties [19]. Recent studies have also investigated the potential health benefits of polyphenols in the prevention and treatment of various diseases. For instance, some studies have suggested that polyphenols may help improve cardiovascular health by reducing inflammation, improving lipid metabolism, and lowering blood pressure. They promote overall health and minimize the risk of diseases associated with unhealthy dietary practices [20]. Other studies have investigated the potential neuroprotective effects of polyphenols in preventing or treating neurodegenerative diseases such as Alzheimer’s and Parkinson’s disease.

In addition to their numerous health benefits, polyphenols have been found to have a positive impact on skin health. These bioactive compounds offer protective and beneficial effects for the skin through various mechanisms. Polyphenols act as potent antioxidants, guarding the skin against the harmful effects of free radicals. By neutralizing these unstable molecules, they help prevent premature aging signs [21]. Certain polyphenols demonstrate anti-inflammatory properties, which can effectively reduce skin inflammation. This can be especially beneficial for individuals dealing with skin conditions like acne or dermatitis, as it helps soothe and calm irritated skin [22]. Furthermore, resveratrol found in red wine has shown photoprotective effects [23] and other polyphenols have the ability to protect the DNA of skin cells from damage caused by external factors [24,25]. Moreover, these compounds contribute to collagen stimulation, a crucial protein responsible for skin structure and elasticity [26]. Polyphenols have been identified as inhibitors of collagen-degrading enzymes, which helps preserve the skin’s texture and prevent premature aging. 

Prolonged exposure to solar ultraviolet (UV) radiation is the primary factor contributing to the majority of skin malignancies [27]. UV radiation, particularly UVB (290–320 nm) from the sun’s spectrum, can serve as both a trigger for tumor development [28,29] and a catalyst for its progression by causing damage to vital cellular components like DNA, proteins, and lipids. Providing mice with a water extract of green tea as their exclusive source of drinking water demonstrated a shielding effect against UVB-radiation-induced tumor initiation and promotion [30]. These findings emphasize the strong connection between polyphenols and their ability to provide protection against the harmful effects of sunlight.

Polyphenols have significant pharmacological functions. They possess potent antioxidant and anti-inflammatory properties and have been linked to a reduced risk of chronic diseases such as cancer, cardiovascular disease, and neurodegenerative disorders [15,31]. Ongoing research about such molecules is confirming promising health benefits associated with these natural compounds (Table 1).

## 2. Distribution in the Mediterranean Area

Polyphenols are widely distributed throughout the world, as many plant-based foods are rich sources of these compounds. Their specific distribution varies by region, with some fruits and vegetables being more common in certain geographic areas than others [40,41]. For example, citrus fruits, which are rich in flavanones, are commonly consumed in Mediterranean countries such as Spain, Italy, and Greece. Berries, which are rich in anthocyanins and ellagitannins, are consumed in many regions, including North America, Europe, and Asia. Grapes, which have elevated levels of stilbenoids, are commonly consumed in Mediterranean countries as well as in other regions where wine production is high, such as Italy, France, California, and Argentina. Tea, which contains tannins, is commonly consumed in many parts of the world, including Asia, Europe, and North America. Finally, flaxseed, which is rich in lignans, is commonly consumed in North America and Europe [42,43]. Overall, a wide variety of plant-based foods containing polyphenols are generally available in most parts of the world, and the specific consumption patterns may vary depending on cultural and regional factors.

**Figure 1:**
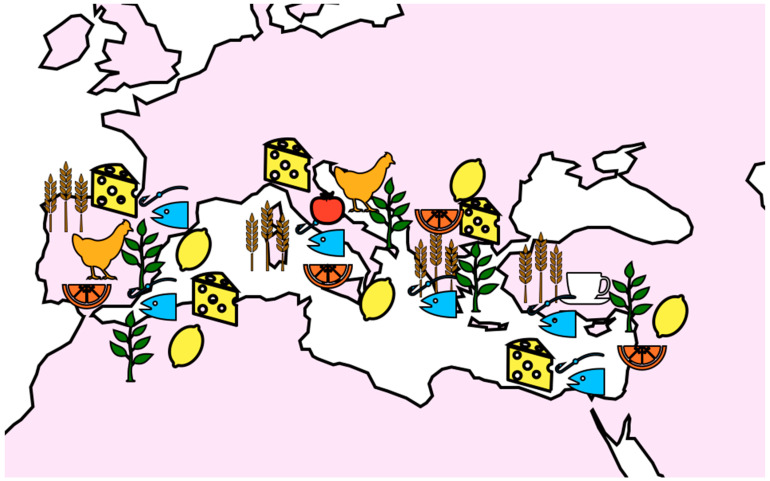
The most consumed foods rich in polyphenols and their distribution in some countries of the Mediterranean area.

The Mediterranean region is a vast area that includes countries such as Spain, Italy, Greece, Turkey, Lebanon, and Morocco as shown in Figure 1. Although the region encompasses a wide variety of cultures and dietary patterns, there are some commonalities in terms of the plant-based foods that are commonly consumed and their polyphenol content.

In Spain, the Mediterranean diet is characterized by a high consumption of fruits, vegetables, nuts, whole grains, fish, poultry, and dairy products. The most commonly consumed fruits in Spain are oranges, mandarins, and lemons, which are rich sources of flavanones such as hesperetin and naringenin. These compounds have been shown to have anti-inflammatory and antioxidant effects, and may contribute to the health benefits of the Mediterranean diet. In addition, Spain is known for its olive oil, which is rich in phenolic compounds such as hydroxytyrosol and oleuropein [35,44]. These compounds have been shown to have a range of health benefits, including anti-inflammatory, antioxidant, and neuroprotective effects.

Italy is the homeland of the Mediterranean diet. Such a regime is characterized by a high consumption of fruits, vegetables, nuts, whole grains, fish, poultry, and milk products. One of the most commonly consumed fruits in Italy is the tomato, which is rich in the polyphenol lycopene. Lycopene has been shown to have antioxidant and anti-inflammatory effects, and may contribute to the health benefits of the Mediterranean diet. In addition, Italy is known for its consumption of red wine, which is rich in polyphenols such as resveratrol. Resveratrol has been shown to have anti-inflammatory, antioxidant, and anti-cancer effects [45,46].

In Greece, the Mediterranean diet is characterized by a high consumption of fruits, vegetables, nuts, and whole grains, as well as fish and dairy products. Some of the most commonly consumed fruits in Greece include oranges, lemons, and pomegranates, which are well-known sources of polyphenols such as hesperetin, naringenin, and ellagitannins. These compounds have been shown to have anti-inflammatory and antioxidant effects, and may contribute to the health benefits of the Mediterranean diet. Greece has in its DNA the production and consumption of olive oil, which is abundant in phenolic compounds such as hydroxytyrosol and oleuropein [47].

Turkey has a main diet characterized by a high consumption of fruits, vegetables, nuts, and whole grains, as well as fish and dairy products. One of the most commonly consumed fruits in Turkey is the fig, which is a copious source of polyphenols such as flavonoids and anthocyanins. These compounds were reported to have anti-inflammatory and antioxidant effects, and may contribute to the health benefits of the Mediterranean diet. In addition, Turkey is known for its consumption of tea, which is rich in catechins [48,49].

In Lebanon, the Mediterranean diet is characterized by a high consumption of fruits, vegetables, nuts, and whole grains, as well as fish, poultry, and dairy products. Some of the most commonly consumed fruits in Lebanon include oranges, lemons, and pomegranates, which are rich sources of hesperetin, naringenin, and ellagitannins. These compounds have been shown to have anti-inflammatory and antioxidant effects, and may contribute to the health benefits of such a diet. Moreover, Lebanon has a great consumption of herbs such as mint and thyme—polyphenol sources too [50,51].

In Morocco, there is a high consumption of fruits, vegetables, nuts, whole grains, fish, and dairy products. Morocco cultivations often include citrus fruits such as oranges and lemons, which are rich sources of hesperetin and naringenin. 

As mentioned above, the Mediterranean diet is mainly a plant-based diet that is rich in polyphenols, which are found in a variety of fruits, vegetables, nuts, and whole grains. The specific types of polyphenols and foods consumed can vary depending on the country and region within the Mediterranean, but the general pattern of a plant-based diet is consistent. The consumption of these foods has been associated with several health benefits, objects of ongoing research, including a lower risk of chronic and inflammatory diseases such as cardiovascular disease and cancer.

### 2.1. Skin Photoprotection

Polyphenols have been extensively studied for their potential photoprotective effects on the skin. Flavonoids, stilbenes, and phenolic acids are some of the most commonly studied polyphenols for their photoprotective effects.

Quercetin is a flavonol that has been shown to possess potent antioxidant properties that can scavenge free radicals and reduce oxidative damage caused by UV radiation. Additionally, quercetin has been shown to have anti-inflammatory effects that can reduce the production of pro-inflammatory cytokines in the skin [34]. Kaempferol, another flavonol, has also been shown to possess strong antioxidant and anti-inflammatory effects that can protect the skin from UV-induced damage [52]. Apigenin, a flavone, has been shown to possess similar photoprotective effects to quercetin and kaempferol [32].

Stilbenes are another group of polyphenols found in plants. The most well-known stilbene is resveratrol, which is found in grapes and red wine. Resveratrol has been shown to have potent antioxidant properties that can scavenge free radicals and reduce oxidative damage caused by UV radiation. Additionally, resveratrol has been shown to activate sirtuins, a class of proteins that are involved in regulating cellular metabolism and stress responses. Sirtuins have been implicated in the regulation of cellular responses to UV radiation and may contribute to the photoprotective effects of resveratrol [53].

Phenolic acids are another class of polyphenols that have been shown to possess photoprotective effects. Caffeic acid and ferulic acid are two of the most commonly studied phenolic acids. Caffeic acid has been shown to have potent antioxidant properties that can reduce oxidative damage caused by UV radiation. Additionally, caffeic acid has been shown to inhibit the production of pro-inflammatory cytokines in the skin. Ferulic acid, another phenolic acid, has also been shown to have potent antioxidant and anti-inflammatory effects that can protect the skin from UV-induced damage. Additionally, ferulic acid has been shown to enhance the efficacy of other antioxidants, such as vitamins C and E, by stabilizing them and improving their ability to scavenge free radicals [37].

Carotenoids are another group of dietary compounds that have been shown to possess photoprotective effects. They are pigments found in a variety of fruits and vegetables, including tomatoes, carrots, and leafy greens. Carotenoids possess potent antioxidant and anti-inflammatory properties that can reduce oxidative damage and inflammation caused by UV radiation. Lycopene, a carotenoid found in tomatoes, has been shown to possess particularly strong photoprotective effects [54].

Green tea, a beverage made from the leaves of the Camellia sinensis plant, is another dietary compound that has been shown to possess photoprotective effects. Green tea contains a class of polyphenols known as catechins, which have been shown to possess strong antioxidant and anti-inflammatory properties. Catechins have been shown to scavenge free radicals and reduce oxidative damage caused by UV radiation [55]. Additionally, they have been shown to inhibit the production of pro-inflammatory cytokines in the skin.

Several studies have investigated the specific mechanisms underlying the photoprotective effects of polyphenols. For example, one study found that quercetin and kaempferol can reduce the expression of matrix metalloproteinases (MMPs) in human skin cells exposed to UV radiation. MMPs are enzymes that degrade collagen and elastin in the skin, leading to wrinkles and sagging. By reducing the expression of MMPs, quercetin and kaempferol may help to maintain the integrity and elasticity of the skin. Another study found that resveratrol can activate the Nrf2/ARE pathway in human skin cells, leading to the upregulation of antioxidant and detoxification enzymes [56,57]. This pathway is an important cellular defense mechanism that protects cells against oxidative stress and may contribute to the photoprotective effects of resveratrol. Overall, polyphenols have been shown to possess a variety of photoprotective properties that can protect the skin from UV-induced damage. These compounds possess potent antioxidant and anti-inflammatory properties that can scavenge free radicals, reduce oxidative damage, and inhibit the production of pro-inflammatory cytokines in the skin. Additionally, some polyphenols have been shown to activate cellular defense mechanisms that can enhance the skin’s ability to cope with UV radiation. By consuming foods rich in polyphenols, individuals may be able to reduce their risk of developing skin damage and premature aging caused by UV radiation [21,58]. Another study by Elmet et al. investigated the effects of green tea polyphenols on skin photoprotection [59]. In order to determine if the green tea polyphenolic fraction (GTPs) could inhibit the UV radiation—an induced erythema response—0.2 mL of GTP in concentrations was applied to the skin. Thirty minutes after the application of the preparation, the subjects were exposed to a 2-MED dose of UV radiation from a solar simulator. Measurements of the erythema response were quantified with a chromameter 24, 48, and 72 h after solar radiation exposure. There was a dose-dependent reduction in erythema with a 10% solution producing almost complete protection at 48 and 72 h. It was demonstrated that a 2.5% solution was found to provide excellent protection, and GTPs were able to produce a significant reduction in the sunburn response. The study found that topical application of green tea polyphenols significantly reduced UV-induced erythema and improved skin barrier function. The researchers suggested that GTPs exert a photoprotective effect on human skin.

According to research conducted by Won et al., the exposure to atmospheric particulate matter (PM10) can lead to skin damage due to cytotoxicity and inflammation. The study also reported that polyphenols from Ecklonia cava, a type of brown algae commonly found in East Asian coastal waters, could help in reducing oxidative stress in keratinocytes of the epidermis that are exposed to PM10 [60]. The extract of E. cava was found to decrease the expression of COX-1, COX-2, and mPGES-2 genes that are stimulated by PM10. E. cava could alleviate the production of PGE2 caused by PM10 in keratinocytes by inhibiting the expression of COX-1, COX-2, mPGES-1, and/or mPGES-2 genes.

Polyphenols demonstrated having both anti-inflammatory and antioxidant effects. These two characteristics made them ideal nutraceuticals for treating and preventing both photo-exposure damage and skin pathological conditions. We decided to focus our attention on the potential of polyphenols in skin photoprotection in atopic dermatitis, psoriasis, and chronic urticaria due to their immune-related pathogenesis and literature available data.

### 2.2. Atopic Dermatitis

Atopic dermatitis (AD) is a chronic inflammatory skin disorder that affects millions of individuals worldwide. The precise underlying mechanisms of AD remain elusive, but factors such as genetics, immunological responses, and environmental elements have been implicated, along with disruptions in skin barrier function [61]. Recent advancements in our comprehension of AD’s pathogenesis have emerged with the discovery of its connection to loss-of-function mutations in filaggrin, a crucial epidermal protein for maintaining skin barrier integrity [62]. These mutations lead to an inheritable dysfunction in the epithelial barrier, facilitating the entry of allergens and microbes. This can trigger a skewed TH2 lymphocyte response, ultimately culminating in eczema [63]. Nonetheless, it has also been proposed that TH2 cytokines may impact barrier function by regulating the expression of filaggrin and other essential structural proteins and peptides crucial for microbial barrier integrity [64]. Regardless of whether they hold a primary or secondary role in the compromised barrier function in AD, it is well-established that T cells play a significant part in the pathological mechanisms of AD. Patients with AD often have an impaired skin barrier function, making them more susceptible to skin damage from environmental factors such as UV radiation. Polyphenols have been investigated for their potential to protect the skin in patients with AD [65,66].

Takatsugu K. et al. conducted a study illustrating the impact of apple polyphenols on individuals suffering from AD. Their research revealed a decrease in inflammation, cracking, itching, sleep disruption, and peripheral blood eosinophil counts among the patients. Additionally, the study demonstrated that administering a daily supplement of apple condensed tannins, at a dosage of 10 mg/kg, significantly ameliorated the symptomatic issues experienced by individuals with AD [67].

Resveratrol is a naturally occurring polyphenol that can be found in various fruits and vegetables, particularly in red grapes and berries. Its beneficial properties include anti-cancer, antioxidant, anti-angiogenic, and anti-inflammatory effects [68,69]. Studies have shown that resveratrol can effectively reduce inflammation and histological changes in a murine model induced with DNFB. It achieves this by modulating apoptosis and regulating cytokine secretion in the epithelium [70]. Based on these findings, Kang et al. conducted a study reporting that the topical application of resveratrol-enriched rice (RR), a genetically modified rice variety that combines the synergistic effects of resveratrol (RSV) and rice, could alleviate skin barrier dysfunction and pruritus in DNCB-treated NC/Nga mice [23]. This finding was completed with a decrease in the expression of pro-inflammatory epithelium cytokines, resulting in reduced cytotoxicity. Notably, among the tested varieties, RR demonstrated the strongest anti-inflammatory, skin repair, and antipruritic effects, showcasing the combined benefits of resveratrol and NR. Consequently, RR appears to offer advantages over RSV and NR, with minimal side effects. In light of these discoveries, the authors propose RR as a potential alternative therapy for managing the pruritic and inflammatory effects of AD.

Overall, these studies suggest that polyphenols may have potential in protecting the skin of patients with AD from UV-induced damage and improving skin barrier function. However, further research is needed to confirm these findings and to identify the optimal dosing and formulation of polyphenols for use in photoprotection and AD treatment.

Atopic dermatitis is a complex disease with a multifactorial pathogenesis, including genetic and environmental factors. The immune response plays a crucial role in the development and progression of atopic dermatitis, with an imbalance of T helper (Th) 1 and Th2 cells leading to increased inflammation. Polyphenols have been studied for their potential to alleviate atopic dermatitis symptoms, as they possess antioxidant and anti-inflammatory properties that may help modulate the immune response. Green tea polyphenols, for example, have been found to suppress the inflammatory response in skin cells and reduce the severity of atopic dermatitis symptoms in mice. In one study, mice with atopic dermatitis were treated with green tea polyphenols, and the researchers found that the polyphenols reduced the expression of pro-inflammatory cytokines and chemokines in the skin, as well as reducing the severity of atopic dermatitis symptoms [71]. A pomegranate extract can reduce the expression of pro-inflammatory cytokines in skin cells and improve the skin barrier function, which is often impaired in individuals with atopic dermatitis [72,73]. These findings suggest that polyphenols may hold promise for the management of atopic dermatitis.

Polyphenols have been shown to have anti-inflammatory effects by inhibiting the production of pro-inflammatory cytokines such as IL-4, IL-5, and IL-13. Additionally, polyphenols have been shown to reduce the expression of IgE receptors on mast cells, which can decrease the binding of IgE antibodies and subsequently reduce the release of histamine and other inflammatory mediators. Polyphenols such as quercetin and epigallocatechin-3-gallate (EGCG) have also been shown to modulate the function of dendritic cells, which play a key role in the initiation of the Th2 immune response [38,39]. As AD usually ameliorates during summer, the application of topical polyphenol-rich products or the intake of polyphenols–nutraceuticals could let AD patients have a better sun-exposure-accelerating skin lesion recovery.

### 2.3. Psoriasis

Psoriasis is a chronic inflammatory disease characterized by hyperproliferation of keratinocytes, and immune system dysregulation. Psoriasis involves various cell types such as Langerhans cells, keratinocytes, endothelial cells, and monocytes. Among these, T cells play a pivotal role in driving inflammation, with T-helper1 (Th1) cells considered the primary instigator of the disease. Psoriasis is characterized by persistent inflammation, resulting in unregulated growth and impaired differentiation of keratinocytes. Examining the histology of psoriatic plaques reveals epidermal thickening covering inflammatory infiltrates comprising dermal dendritic cells, macrophages, T cells, and neutrophils as mentioned above. Notably, neovascularization is a prominent aspect of this condition [74]. The inflammatory response is a consequence of the collaborative action of Th1 and Th17 cells. The discovery of Th17 and Th22 cells, along with their associated cytokines like IL-22 and IL-23, has provided promising targets for the development of novel pharmaceuticals. Th17 cells produce cytokines like IL-17A, IL-17F, and IL-22, which lead to the proliferation of keratinocytes [75]. Additionally, they stimulate the production of tumor necrosis factor-α (TNF-α), chemokine ligand (CXCL) 1, and CXCL8 [76]. TNF-α, in turn, expedites the infiltration of inflammatory cells (such as lymphocytes, monocytes, and neutrophils) into the skin layers. Mediators released by macrophages, keratinocytes, Langerhans cells (a type of dendritic cell), and T cells stimulate the proliferation of keratinocytes. Keratinocytes themselves generate a range of cytokines and growth factors that contribute to both inflammation and proliferation. Mast cells produce TNF-α, interferon γ (IFN-γ), IL-8, and VEGF, resulting in an increased presence of T cells and neutrophils at the inflammation site. TNF-α triggers the activation of the NF-κB signaling pathway, which in turn governs the survival, proliferation, and anti-apoptotic effects of both keratinocytes and lymphocytes [77]. Furthermore, recent research [78] has also highlighted the potential involvement of IL-33 in the activation of keratinocytes. Specifically, it was demonstrated that the combination of TNFα and INFγ leads to the expression of IL-33, subsequently resulting in the suppression of IL-8 activity [79]. In a separate study by the same authors [80], it was found that IL-17A may enhance the expression of IL-33 in an NHEK culture, likely through the induction of ERK, p38/MAPK, and JAK/STAT signaling pathways. They also observed that the synergistic effect of IL-17A and TNFα does not lead to the induction of IL-33. 

The disease affects about 2–4% of the global population, and it has been associated with an increased risk of skin cancer, particularly in individuals with severe and longstanding disease [81]. Several studies have investigated the potential role of polyphenols in skin photoprotection in psoriasis.

A study was conducted to assess the impact of applying a green tea extract topically on individuals with psoriasis. A green tea extract contains polyphenols known as catechins, which are found in the leaves of the tea plant (Camellia sinensis). The major polyphenols in green tea leaves are (−)-epicatechin (EC), (−)-epigallocatechin (EGC), (−)-epicatechin-3-gallate (ECG), and (−)-epigallocatechin-3-gallate (EGCG). Among these, EGCG is the most abundant and considered the most potent green tea polyphenol (GTP) [82].

In the study, EGCG was topically applied to normal human epidermal keratinocytes (NHEKs), which are rapidly growing skin cells. This application demonstrated potential benefits of GTPs for psoriasiform lesions. The findings also revealed that the expression of caspase 14 in NHEK induced using EGCG is dependent on the p38 and JNK MAPK pathways. Notably, when 0.5% GTPs were applied topically, significant improvements in epidermal pathology symptoms were observed in mice with flaky skin. This improvement was associated with effective caspase 14 processing and a reduction in levels of the proliferating cell nuclear antigen.

Furthermore, red wines are believed to possess beneficial nutritional effects due to the presence of trace amounts of resveratrol [83,84]. Additionally, various studies have indicated that applying a cream containing resveratrol, a polyphenol present in grapes and red wine, can have positive effects on skin lesions in individuals with psoriasis [36,85,86]. 

Some researchers recommend a proper treatment approach involving the topical administration of resveratrol at concentrations ranging from 0.01% to 20%, preferably 1% to 5%, in the form of lotions, creams, or ointments [87]. This treatment may also be combined with other active ingredients like melatonin and vitamins D, E, and A, or their derivatives. A double-blind study revealed that 80% of patients treated with the resveratrol-containing ointment experienced significant improvement, in contrast to only 10% in the control group. Furthermore, the combination of resveratrol and vitamin D derivatives was effective in 95% of patients. The study concluded that patients using the resveratrol cream exhibited noteworthy enhancements in skin hydration and overall quality of life, suggesting anti-inflammatory properties. Moreover, the severity of psoriasis lesions was found to be reduced in the resveratrol group compared to the placebo group. Curcumin, an orange-yellow diketone pigment naturally found in turmeric rhizomes, is a plant polyphenol known for its effects on skin disorders. Extensive evidence supports its therapeutic potential in this regard [88,89]. A recent study aimed to investigate the inhibitory effects of curcumin on the NLRP3 inflammatory body and its ability to reduce inflammation in a mouse model of psoriasis [90]. The study employed a curcumin gel for percutaneous administration and utilized the mouse psoriasis model to examine the mechanisms underlying curcumin’s anti-inflammatory properties. To further evaluate the therapeutic effects of curcumin on psoriasis, the researchers established a control group and an experimental group. The findings indicate that curcumin effectively inhibits NLRP3 inflammatory bodies, thereby reducing the expression of NLRP3 and suppressing IL-22- and IL-18-induced inflammation. Furthermore, curcumin minimizes the damage caused by psoriasis, as it significantly inhibits STAT3 phosphorylation induced with IL-22, achieving a reduction of 95.6%, and exhibits a 47% inhibition of IL-22 and IL-18.

Several polyphenols have been discovered in Amphipterygium adstringens, including gallic acid, which is a polyphenolic compound that occurs naturally in various plants. Gallic acid possesses antioxidant, anti-inflammatory, and antimicrobial properties [91]. A recent study successfully conducted a physicochemical analysis of pyrolytic fractions obtained from the bark of A. adstringens, a plant traditionally used in Mexican folk medicine to treat skin conditions [92]. By utilizing a combination of analytical techniques, the study provided an overview of the molecules present in pyrolytic oils and residual biomass. The pyrolytic oils demonstrated significant inhibitory effects on the production of IL-8, suggesting their potential for treating IL-17-driven dermatological diseases like psoriasis. Notably, oil-2 at a concentration of 15 μg/mL exhibited a similar inhibitory effect on IL-8 as dexamethasone, a commonly used drug for managing this disease. Additionally, the study revealed that the residual biomass from A. adstringens bark, typically considered waste, contains valuable bioactive compounds. Pyrolysis of the biomass after extraction offers an interesting alternative for utilizing this waste to develop new therapeutic candidates with high value. However, further research is required before establishing the therapeutic use of pyrolytic oils from A. adstringens bark for psoriasis. Specifically, identifying and quantifying the molecules responsible for the anti-inflammatory activity and investigating their impact on IL-17-related pathways are crucial aspects that need to be explored.

The pathogenesis of psoriasis involves an overactive immune response, leading to the proliferation of skin cells and the production of inflammatory cytokines. Polyphenols have been investigated for their potential to alleviate psoriasis symptoms, as they possess anti-inflammatory and immunomodulatory properties that may help regulate the immune response. Curcumin, a polyphenol found in turmeric, has been found to reduce the thickness of psoriatic skin lesions and reduce the expression of inflammatory markers in the skin in animal studies [90]. Resveratrol has been found to inhibit the activation of immune cells in the skin, reducing the severity of psoriasis symptoms in mice [23,68,87]. These findings suggest that polyphenols may be a promising avenue for the treatment of psoriasis. Polyphenols have been shown to modulate the immune response in psoriasis by inhibiting the production of pro-inflammatory cytokines such as TNF-α, IL-6, and IL-17. Additionally, polyphenols have been shown to inhibit the activation of nuclear factor kappa B (NF-κB), a transcription factor that regulates the expression of genes involved in inflammation. Resveratrol and curcumin have also been shown to inhibit the proliferation and activation of T cells. 

Hence, the immunomodulatory effects of phenolic components also exhibit their positive influence on the immune response, indicating that certain nutrients have the capacity to regulate miRNAs in psoriasis. Specifically, miR-203, primarily found in keratinocytes, has shown abnormal expression in psoriasis. This aberration has been linked to various outcomes, including heightened levels of STAT3 (signal transducer and activator of transcription) protein; increased activity of protein kinase C (a vital regulator of keratinocyte differentiation); enhanced transcription of pro-inflammatory cytokines like TNF-α, IL-8, and IL-24; reduced expression of the suppressor of cytokine-signaling SOCS3 protein (which binds to tyrosine kinase receptors and the JAK/STAT proliferative pathway); and decreased transcription factor p63 (a squamous epithelia differentiation-associated protein and part of the p53-related protein family). In summary, specific nutrients such as polyphenols possess the ability to modulate miRNA expression patterns, suggesting a potential nutrigenomic profile that may benefit individuals with psoriasis [93]. As it is known, patients affected by psoriasis could benefit from UV treatments. The use of topic or systemic polyphenolic nutraceuticals could help in avoiding detrimental sunlight effects and leave only the beneficial ones.

### 2.4. Chronic Urticaria

Chronic urticaria (CU) is a skin disease characterized by recurrent itchy wheals, angioedema, or both, lasting for more than 6 weeks. The disease pathogenesis involves the activation of mast cells and the release of histamine and other inflammatory mediators [94]. In addition, oxidative stress and reactive oxygen species (ROS) have also been reported to be involved in the pathogenesis of CU [95,96]. More recently, there has been growing experimental and clinical evidence supporting the autoimmune etiology in numerous cases of CU [97,98]. It is estimated that around 45% of patients with CU have an autoimmune basis. In a variable range, spanning from 30% to 60% of patients exhibiting active disease, the intradermal administration of autologous serum (commonly known as the autologous serum skin test, ASST) elicits a wheal-and-flare reaction. Additionally, the serum obtained from certain CU patients has the capacity to prompt histamine release from cultured basophils of healthy individuals. These phenomena have been attributed to the presence of circulating IgG antibodies that specifically target the high-affinity IgE receptor FceRI found on mast cells and basophils, or alternatively, IgE [99]. These IgG antibodies set off the classical complement cascade [100]. Upon subclass purification of IgG, it has been observed that histamine-releasing activity is predominantly associated with subclasses 1 and 3 [101]. Other autoantibodies may also play a contributory role in CU. A recent extensive in vitro investigation identified autoantibodies against CD23, which is the low-affinity IgE receptor, in as many as 65% of CU patients [102]. This particular autoantibody can induce mast cell degranulation indirectly in vitro by triggering the release of the major basic protein from eosinophils. The frequent co-occurrence of CU with antithyroid antibodies, recognized markers of autoimmunity, further bolsters the autoimmune nature of this condition. 

Several studies have demonstrated that polyphenols, especially flavonoids, possess antioxidant properties and can reduce oxidative stress [103,104]. Therefore, polyphenols may play a role in the prevention and management of CU by reducing oxidative stress and mast cell activation [105].

A resveratrol extract demonstrated promising results on mast cell activation. The study investigated the effect of resveratrol in regulating MRGPRX2-mediated MC activation and its underlying mechanism. Resveratrol inhibited MCs’ degranulation in vitro. Resveratrol also suppressed extravasation, active systemic anaphylaxis, and MCs’ degranulation in mouse models of pseudo-allergy in vivo. The study supports the potential application of resveratrol nutraceuticals in CU patients [106]. 

In another study, a total of 153 patients diagnosed with chronic urticaria (CU) were involved. These patients adhered to a diet free of pseudoallergens and rich in polyphenols. The findings revealed that complete remission of symptoms was observed in the patients who strictly avoided the identified trigger foods. Thus, the implementation of an incremental build-up food challenge (IBUF) protocol presents a viable approach for CU patients to achieve enduring symptom relief through a straightforward dietary regimen. The outcomes demonstrated a significant enhancement in symptoms for the group that followed the pseudoallergen-free and rich-in-polyphenols diet for a duration of 5 weeks. Moreover, subjective disturbance and overall quality of life were notably improved [107]. 

In a recent review, the potential utilization of quercetin and luteolin in managing various diseases, including CU, was discussed [33]. The authors emphasized numerous studies that highlighted the antioxidant and anti-inflammatory properties of polyphenols, as well as their potential in reducing oxidative stress and mast cell activation in the skin [108,109]. Furthermore, the authors also suggested that combining polyphenols with other treatments, such as antihistamines, could potentially lead to improved outcomes for CU patients [110].

The available evidence suggests that polyphenols may play a beneficial role in the management of chronic urticaria by reducing oxidative stress and inflammation in the skin. However, further studies are needed to better understand the mechanisms underlying these effects and to determine the optimal dosages and formulations of polyphenols for CU co-treatment.

**Figure 2:**
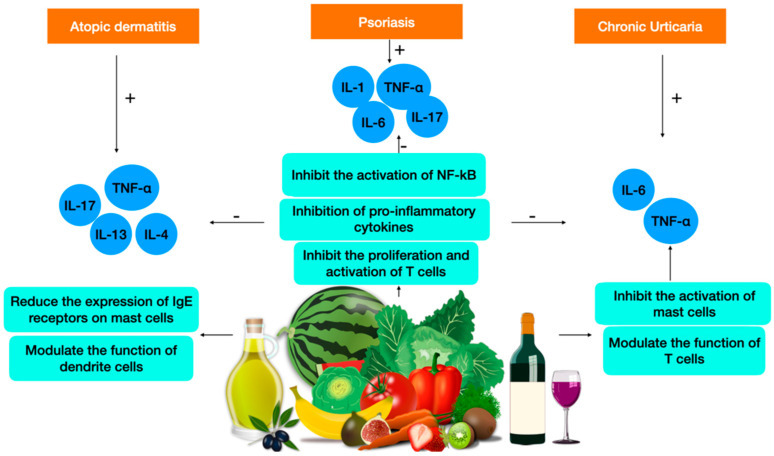
Polyphenols from foods of the Mediterranean area and their role in atopic dermatitis, psoriasis, and chronic urticaria.

The pathogenesis of chronic urticaria involves the activation of mast cells, leading to the release of histamine and other inflammatory mediators. Polyphenols have been studied for their potential to alleviate chronic urticaria symptoms, as they possess anti-inflammatory and antioxidant properties that may help reduce the activation of mast cells. Polyphenols have been found to reduce the severity of hives and decrease the expression of inflammatory cytokines. Polyphenols have been shown to have anti-inflammatory effects in CU by inhibiting the production of pro-inflammatory cytokines such as IL-6 and IL-8. Additionally, polyphenols have been shown to inhibit the activation of mast cells, which play a key role in the pathogenesis of chronic urticaria. Some of these intriguing molecules have also been shown to modulate the function of T cells, which may contribute to the immune dysregulation. Overall, the immunomodulatory effects of polyphenols may provide a promising add-on therapeutic approach for the treatment of chronic urticaria (Figure 2). Their potential for add-on therapy together with their ability to reduce inflammation and ROS effects could also ameliorate sunlight-related detrimental effects.

## 3. Conclusions and Future Perspectives

In conclusion, polyphenols and their reported pharmacological effects highlight the importance of exploring natural compounds for therapeutic purposes. The wide variety of polyphenols found in different foods and plants allows for a diverse range of pharmacological effects, and their potential use in treating various dermatological conditions is a promising area of research. The Mediterranean diet, rich in polyphenol-containing foods, has been associated with a lower incidence of various chronic diseases, including dermatological conditions. While more research is needed to fully understand the mechanisms of action and optimal dosing of polyphenols, there is initial evidence to support their potential use as adjunctive therapy for conditions such as atopic dermatitis, psoriasis, and chronic urticaria, especially for their photoprotective properties. 

Furthermore, the use of polyphenols as nutraceuticals may offer several advantages over traditional treatments, including fewer adverse effects and a more natural approach to disease management. In fact, the available evidence suggests that polyphenols may play a beneficial role in the management of psoriasis, atopic dermatitis, and chronic urticaria by reducing oxidative stress and inflammation in the skin. Their employment could be both as topical medicaments to be applied on skin lesions and as systemic medicaments in order to prevent the onset of the disease or to accelerate the recovery. As far as we know, the only disadvantage of such treatments could be due to a patient not responding to the nutraceutical or to an adverse effect against the polyphenol source. However, the field of polyphenol research is still in its early stages, and more randomized controlled trials are needed to fully assess the efficacy and safety of these compounds in clinical settings. In addition, there is a need for more research into the specific mechanisms of action of different polyphenols and how they can be optimized for specific dermatological conditions. This could involve exploring the use of combination therapies, personalized medicine approaches, and the development of novel drug delivery systems to enhance the bioavailability and efficacy of polyphenols. 

Overall, the study of polyphenols and their potential therapeutic applications for some dermatological conditions is an interesting area of research that holds promise for improving patient outcomes and reducing the burden of disease. 

## Figures and Tables

**Table 1:** List of the main polyphenols with their natural sources and effects on health.

Polyphenol Class	Examples	Dietary Sources	Pharmacological Functions	References
Flavonoids	Apigenin, Luteolin, Quercetin, Kaempferol	Fruits, vegetables, nuts, and herbs	Anti-inflammatory, antioxidant, anti-cancer, improved cardiovascular health	[7,32,33,34]
Flavanones	Hesperetin, Naringenin	Citrus fruits	Anti-inflammatory, antioxidant, improved insulin sensitivity, reduced risk of cardiovascular disease	[10,35]
Anthocyanins	Cyanidin, Delphinidin, Pelargonidin	Red, purple, and blue fruits and vegetables	Anti-inflammatory, antioxidant, improved cognitive function, reduced risk of cardiovascular disease	[10,11,36]
Stilbenoids	Resveratrol	Grapes, berries, and nuts	Anti-inflammatory, antioxidant, anti-cancer, improved insulin sensitivity, neuroprotective	[12,14,23]
Phenolic acids	Caffeic acid, Rosmarinic acid	Fruits, vegetables, and herbs	Anti-inflammatory, antioxidant, reduced risk of chronic diseases	[16,37]
Lignans	Secoisolariciresinol, Matairesinol	Flaxseed, sesame seeds, and whole grains	Anti-inflammatory, antioxidant, reduced risk of breast and prostate cancer, improved insulin sensitivity	[17]
Tannins	Catechins, Epicatechins	Tea and wine	Anti-inflammatory, antioxidant, potential anti-cancer effects	[18,38,39]
Ellagitannins	Punicalagin, Granatin B	Berries and nuts	Anti-inflammatory, antioxidant, potential anti-cancer effects	[18,19]

## Data Availability

Not applicable.
